# A Novel Low-Cost Uroflowmetry for Patient Telemonitoring

**DOI:** 10.3390/ijerph20043287

**Published:** 2023-02-13

**Authors:** Savio Domenico Pandolfo, Federica Crauso, Achille Aveta, Simone Cilio, Biagio Barone, Luigi Napolitano, Antonio Scarpato, Benito Fabio Mirto, Francesco Serino, Francesco Del Giudice, Benjamin I. Chung, Fabio Crocerossa, Erika Di Zazzo, Francesco Trama, Ruggero Vaglio, Zhenjie Wu, Paolo Verze, Ciro Imbimbo, Felice Crocetto

**Affiliations:** 1 Department of Neurosciences, Reproductive Sciences and Odontostomatology, University of Naples “Federico II”, 80131 Naples, Italy; 2 Division of Urology, Virginia Commonwealth University (VCU) Health, Richmond, VA 23298, USA; 3 Department of Public Health, University of Naples “Federico II”, 80125 Naples, Italy; 4 NexsusTLC SRLS, 80010 Quarto, Italy; 5 Department of Maternal-Infant and Urologic Sciences, “Sapienza” University of Rome, Policlinico Umberto I Hospital, 00161 Rome, Italy; 6 Department of Urology, Stanford Medical Center, Stanford, CA 94305, USA; 7 Urology Unit, Magna Graecia University of Catanzaro, 88100 Catanzaro, Italy; 8 Department of Medicine and Health Sciences “V. Tiberio”, University of Molise, 86100 Campobasso, Italy; 9 Andrological and Urogynecological Clinic, Santa Maria Terni Hospital, University of Perugia, 05100 Terni, Italy; 10 Department of Physics, University of Naples “Federico II”, and CNR SPIN, 80125 Naples, Italy; 11 Department of Urology, Changhai Hospital, Naval Medical University, Shanghai 200433, China; 12 Department of Medicine and Surgery, Scuola Medica Salernitana, University of Salerno, 84081 Fisciano, Italy

**Keywords:** uroflow, home uroflowmetry, urodynamics, software, BPH

## Abstract

Uroflowmetry (UF) is a crucial guideline-recommended tool for men with benign prostatic obstruction (BPO). Moreover, UF is a helpful decision-making tool for the management of patients with lower urinary tract symptoms (LUTS) and benign prostatic hyperplasia (BPH). In the last few years, telemedicine and telehealth have increased exponentially as cost-effective treatment options for both patients and physicians. Telemedicine and telehealth have been well positioned during the COVID-19 pandemic to prevent healthcare system overload and to ensure adequate management of patients through screening, diagnosis, and follow-up at home. In the present manuscript, the main characteristics and performance of a novel and low-cost device for home-based UF have been analyzed. The simple weight-transducer method has been applied to perform UF. An inexpensive load cell connected to a 24 bit analogic digital converter (ADC) sends data to a cloud server via SIM card or home Wi-Fi. Data are processed and shown in graphics with both volume and flow rate as a function of time, allowing for measurement of average flow rate, maximum flow rate, voided volume, and voiding time. A numerical algorithm allows for filtering of the dynamic effect due to the urine gravity acceleration and for removing the funnel to simplify the home measurement procedure. Through an online platform, the physician can see and compare each UF data. The device’s reliability has been validated in a first laboratory setting and showed excellent performance. This approach based on domiciliary tests and an online platform can revolutionize the urologic clinic landscape by offering a constant patient cost-effective follow-up, eliminating the time wasted waiting in the office setting.

## 1. Introduction

Uroflowmetry (UF) is a crucial guideline-recommended tool for men with benign prostatic obstruction (BPO) and is a helpful decision-making tool for patients with lower urinary tract symptoms (LUTS) and benign prostatic hyperplasia (BPH) [1]. 

Requirements for UF described by a 2018 International Continence Society (ICS) Standardization Report include adequate privacy, a normal desire to void, and repeated and representative measurements. The variables measured should include a graphical plot of flow rate against time for the whole void, maximum urinary flow rate (Qmax), voided volume (Vvoid), and post-void residual volume (PVR) [2]. Current UF is an office-based procedure requiring “on-demand” urination. Most clinicians use a single office measurement of flow rate, but this approach may not be ideal because of physiological and circadian variations in urination [3]. For this reason, single-test office UF cannot be considered a universally reliable procedure for BPO determination; it has been reported that some patients are unable to relax in unfamiliar environments [4]. Although multiple office readings are time and cost-consuming, they have been performed to avoid these limitations and to improve diagnostic accuracy [5]. Therefore, consecutive tests are recommended to obtain accurate measurements because of variability in several voiding parameters [6]. Another possibility is a home-based UF device that enables multiple measurements in line with an individual’s day-to-day voiding habits and could potentially be used as part of the assessment of men with LUTS. For this reason, a comfortable, friendly, and portable home-based UF may overcome office-based UF limits. 

Over time, different home devices have been projected for the purpose, however, in real-world settings their application was poor and none of them offer an online telemonitoring platform (CIT). Moreover, the COVID-19 pandemic has considerably challenged the healthcare system worldwide, resulting in the impossibility of providing timely and effective diagnoses and treatments [5]. For this reason, the European Association of Urology (EAU) guidelines on male LUTS and BPH have recommended telemedicine for assessment and follow-up in the COVID-19 era and beyond [1,6].

In this scenario, one of the more appealing strategies could be a low-cost home-based UF as a useful urodynamic tool considering social distancing measures. Moreover, the use of a home-based UF in a private setting could overcome the office-based UF limits considering its advantages of decreasing variability through the statistical benefit of averaging multiple measurements and complying with circadian rhythms.

Herein, we show our novel low-cost UF describing physical principles, device configuration, and data acquisition.

## 2. Methods

The developed device has been recorded with the following patent number: 102021000032333.

### 2.1. Physical Principles of the Flow Measurement

**Figure 1:**
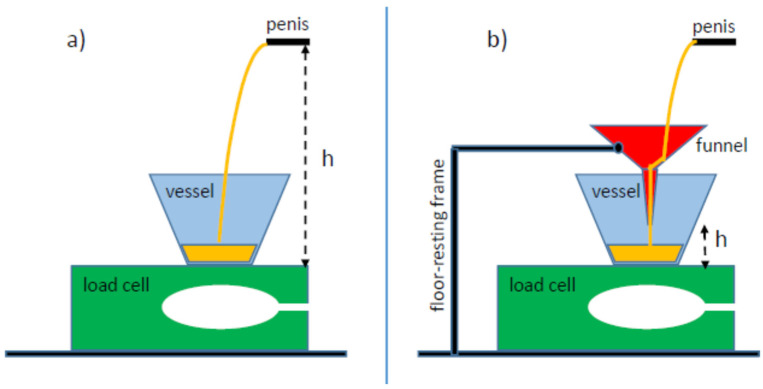
Simplified configuration adopted in our work and the classical configuration. (**a**) This represents our home-based UF. The load cell measures the force exerted by the urine weight deposited on a vessel located on the cell. Our device is more comfortable and user-friendly than (**b**) the standard office-based device, which requires a funnel between the penis and the vessel.

Different methods can be used for measuring urine flow and in general any liquid flow. The load cell or weight transducer method represents the simplest and the most used technique [7]. When using the load cell method, it is customary to insert a funnel between the penis and the vessel, connected to a frame on the floor. This represents the standard office-based UF, which although reliable, would be uncomfortable and unfriendly in a home setting. To this purpose, we developed a new comfortable and user-friendly home-based UF by applying a specific algorithm that allowed us to remove the funnel (Figure 1a).

The load cell measures the force exerted by the urine weight deposited on a vessel located on the cell as schematically reported in Figure 1a. The force F measured by the cell is a function of time (t) and can be expressed in the following way [8]:

(1) $$F\left(t\right)=M\left(t\right)g+\frac{dP\left(t\right)}{dt}$$

Here, *M* is the urine mass, *g* the gravity acceleration (9.8 m/s^2^ at sea level) and *P* is the urine momentum (*P* = *Mv*), where *v* is the velocity of the urine when it reaches the bottom of the vessel (the symbol indicates the time derivative of momentum). The urine mass can be written as *M* = *ρV*, where *V* is the urine volume and *ρ* is the urine density. Neglecting the vertical component of the urine emitted at the penis level, the velocity is just the “fall velocity”, where *h* is the distance between the penis and the surface of the urine in the vessel. However, during the process, the level of urine in the vessel increases, and if the vessel is large enough, *h* can be assumed to be the distance between the penis level and the bottom of the vessel. The fall velocity can be assumed constant.

In order to determine *h*, the patient height has to be acquired during the anamnesis process. Then, the distance of the penis from the ground can be assumed to be half of the patient height. The basis of the load cell must be set on the cover of the bathroom cup or on any equivalent surface at a height of about 45 cm from the ground. *H* is the difference between the penis and the support surface heights in respect to ground. This procedure would generate an error on h that can be estimated being on the order of 10%.

We can rewrite Equation (1) in the following way:

$$F\left(t\right)=\rho gV\left(t\right)+\rho \sqrt{2gh}\frac{dV\left(t\right)}{dt}\;\mathrm{or}:\;\frac{F\left(t\right)}{\rho g}=V\left(t\right)+\sqrt{\frac{2h}{g}}\frac{dV\left(t\right)}{dt}$$

The flux (or flow rate) is $R\left(t\right)=\frac{dV\left(t\right)}{dt}$. We can now define the “measured volume” $V_{m}\left(t\right)=\frac{F\left(t\right)}{\rho g}$ by a simple calibration procedure, we can set *V_m_*(*t*) = *V*(*t*) at the end of the voiding process when the flow rate is zero, with the urine volume measured in ml. Since urine density *ρ* changes of less than 4% among different patients (or for the same patients at different times), within the uncertainty limits of the full urine flux measuring procedure, the calibration procedure needs only to be carried out once, before starting clinical use of the device.

Introducing the “time of fall” $\tau =\sqrt{\frac{2h}{g}}$ (that represents the time that the urine takes from emission at the penis level to reach the bottom of the vessel), we can rewrite the final differential equation:

(2) $$V_{m}\left(t\right)=V\left(t\right)+\tau \frac{dV\left(t\right)}{dt}$$

The second term on the right in Equation (2) is sometimes referred to as the “gravity term” and represents a correction term, i.e., the difference between the effective and measured volumes. As already mentioned, when using the load cell method, it is customary to insert a funnel between the penis and the vessel (Figure 1b). This reduces the fall height *h* (and, consequently, the fall time *τ* to very low values, so that the “gravity term” can be fully neglected in Equation (2) and the “measured volume” coincides with the effective urine volume ($V_{m}\left(t\right)=V\left(t\right)\;at\;any\;time$). In this case, the flux can be simply calculated by taking the measured volume derivative. However, in our opinion, for a home-based UF setting, introduction of the funnel makes the device more complex and unpractical for the patients. Consequentially, we removed the funnel relying on the full Equation (2) to obtain the real urine volume and flux from the measurements.

Equation (2) is a differential equation that can be solved numerically by a complex procedure. However, for a fall height *h ≤ 0.9 m* (patient height less than 2.4 m; fall time *τ < 0.4 s*) and any flux rate, the gravity term is small, and a simpler, although approximate, procedure can be safely used. In a first-order approximation, we can estimate the gravity term by initially computing the flux from the measured volume. This introduces just a small error since we are using an approximate volume determination (*V_m_(t)*) only in the correction term. Then and from Equation (2), we can obtain the effective volume from the measured one, using the equation: by inverting Equation (2):

(3) $$V\left(t\right)=V_{m}\left(t\right)-\tau \frac{dV_{m}\left(t\right)}{dt}$$

Then, we obtain the effective, although approximate, flux by $R\left(t\right)=\frac{dV\left(t\right)}{dt}$. This procedure can be simply implemented numerically from the acquired load cell data.

The use of this technique reduces systematic error due to the gravity term. In percentage, the entity of this systematic error is larger for high flux rates and is larger at the first stages of the urination process (when the deposited urine volume is small).

This is quantitatively illustrated in Supplementary Table S1, where the error in percentage due to the gravity term is calculated from Equation (3) for different values of volume, flow, and fall height. The data show how the addition of the second term (gravity term) affects the measurements. We can say that without the algorithm, we would have an error always higher than 8%, exceeding what the guidelines recommend [2], especially for higher flow rates and at the beginning of urination.

### 2.2. Prototype Device

#### 2.2.1. Load Cell, Electronics and Device Design

**Figure 2:**
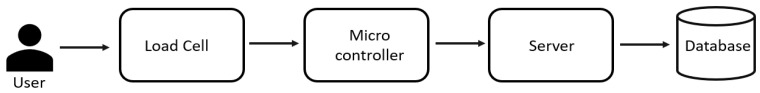
Measurement and data transfer method. The weight transducer, namely the load cell, can detect the urine weight and communicate with the microcontroller, sending the recorded measurements to a dedicated server.

Our UF consists of two main parts: the weight transducer, namely the load cell, that can detect the urine weight and a microcontroller that interfaces with an analog digital converter (ADC) and sends the recorded measurements to a dedicated server (Figure 2). The load cell can appreciate a weight not exceeding 1 kg, and it is equipped with a 24 bit ADC, 80 Hz sampling frequency. The device, due to the SIM800l module, sends data without having to be configured to any Wi-Fi network. The microcontroller sends the data array via Sim card or Wi-Fi connection. The microcontroller is a Node MCU ESP32, a low-cost and powerful system with an integrated Sim card slot, Wi-Fi, and dual-mode Bluetooth. The Flash File System for Serial Peripherical Interface (SPIFFS) allows access to the flash memory to read and write the files. In this flash memory, the ESP 32 stores the files. Moreover, after starting the measurement, the SPIFF records and sends the data to the server at the end of the measurement. The microcontroller is programmed with a maximum of 10 s waiting time to calculate the time delay. The delay time is calculated as the time difference between the beginning of the measurement and the time of the first non-zero acquired datum. Once GPRS (General Packet Radio Service) connectivity is established, the display indicates that the device is ready for performing the measurement.

The system includes a rechargeable battery and two buttons, one to turn on the device, and another to boot/break/stop the measurement. Once the measure has started, the display indicates that the measurement is ongoing, and the urine voided volume is displayed at the end of the measurement. At each measurement, the device is self-resettled to the volume of 0 mL. An acoustic signal is introduced to provide further feedback to the patient about the start and end measurement process, along with the resulting data transmission. If the user forgets to press the stop button after the measurement, the device after 100 acquisitions (20 s) of the same volume value interrupts the operation, and data are sent, always preceded by the acoustic signal feedback, to the server.

**Figure 3:**
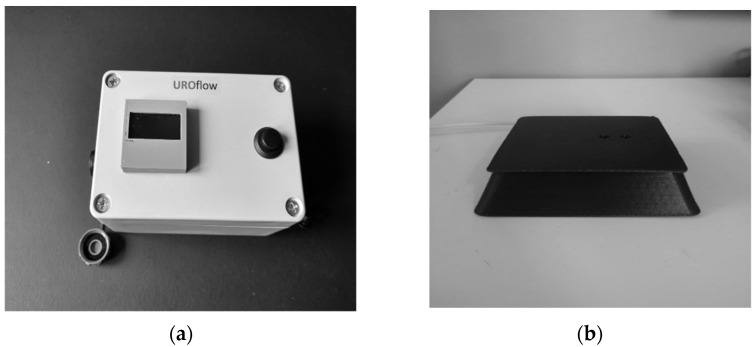

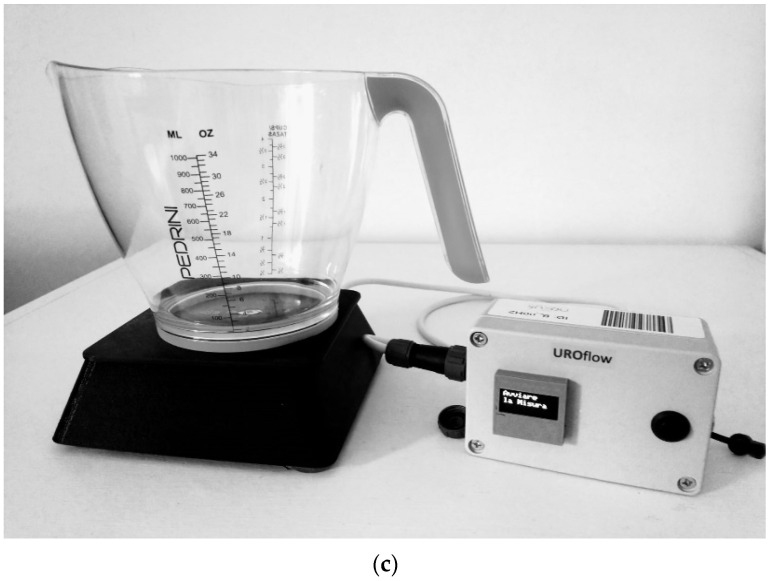
Prototype device: (**a**) electronics; (**b**) load cell [in a 3D-printed case.]; (**c**) setting of the system (standing position).

The device design maintains a separation between the electronics and the load cell to make the device easy to use (Figure 3c). The patient places the load cell included in a 3D-printed case, shown in Figure 3b, on the toilet, and the electronics (Figure 3a) nearby with a maximum distance of 1.5 m. The container used to perform the measurement can be any vessel, but to avoid urine dispersion, the upper diameter should be 15 cm or more and the lower diameter should be about 10 cm, to well accommodate the vessel on the load cell and to guarantee structural stability. The vessel weight should be sufficient for stability (up to a maximum of 200–300 mg).

#### 2.2.2. Data Transmission, Acquisition, and Processing

**Figure 4:**
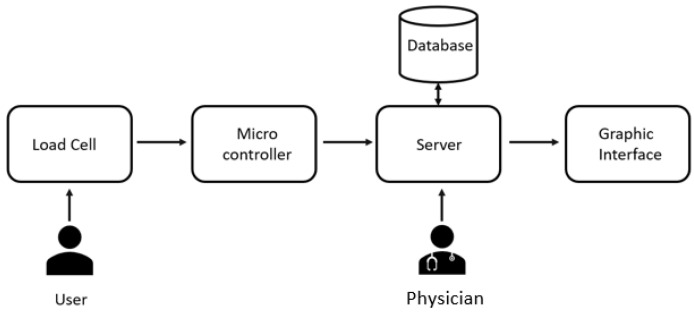
Hardware and software architecture. The load cell communicating with the microcontroller sends the recorded measurements to a dedicated server. The data contain information about urodynamic parameters and device id. This data array is saved in a database associated with a specific patient. The physician can view the recorded measurement.

Data acquisition and processing were designed in accordance with the ICS Guidelines on Urodynamic Performance Equipment [9]. The UF sends a data array to the server, using the HTTPS protocol (Figure 4). The data contain information about urodynamic parameters and device id.

This data array is saved in a database associated with a specific patient and is sent to the physician. Therefore, before delivering the device to the patient, the physician must register the patient and associate the device with the dedicated platform.

The platform is structured in three main parts: the first dedicated to patient registration, the second reserved for the patient chart and device assignment, and a third for graphical visualization. The physician can view the recorded measurement indicating the day and time of interest and the patient’s data.

Moreover, the physician can select several measurements of the same patient to compare them both graphically and numerically. In addition, the system allows for communication with patients through a dedicated APP and deletes unwanted measurements.

## 3. Cost Analysis

The cost analysis was carried out by the innovative PMI Nexus TLC and includes an evaluation of the hardware, software, and server part (Table 1). Consumables for the device were calculate at EUR 68.22 per case (Table 1), the full cost increases considering telemonitoring services offered. This cost is lower compared to competitors for the funnel and kingpost removal, which gives not only user-friendly but also financial profit.

Moreover, as we have developed a physical device and an online service platform, those may be sold separately to offer a diversified service according to the requests. In this view, B2B as well as B2C business is possible. After these considerations, amortized costs to produce 1000 devices were calculated. It was determined that the cost of the full product is about EUR 100. This cost could be further reduced including a monthly cost for telemonitoring services (EUR 5) on the type of adopted business.

Currently, different office-based devices are available, all more expensive compared to ours, with a cost around EUR 1000–3000. This allowed us to present an innovative and low-cost UF.

## 4. Urinary Flow Measurement in a Laboratory Setting

A laboratory measurement was made in order to assess the reliability of the system. The accuracy of this device is assessed by dropping a fluid, with a known flowrate, and by comparing the recorded/measured fluid flowrate with the one poured and known [10]. The flowmeters have accepted results if the volume error is 1–8% and Q errors 4–15% [11,12]. Thirty tests were carried out using a small funnel filled with 200 mL of water, generating a calibrated flow of 4 mL/s.

**Figure 5:**
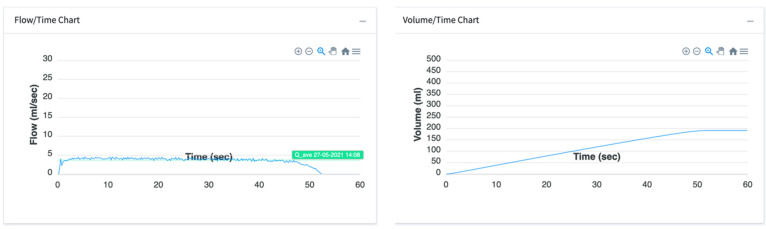
Graphic value report. Urine flow vs. time (s) and variation of urine volume (ml) over time (s).

The following coefficients were collected (Table 2): maximum flow rate (Q_max_), time to void in seconds (T_total_), average flow rate (Q_ave_), voided volume (V_voided_), and delay time. The mean and standard deviation were calculated for all the measurements. The correlation between voided volume and predicted voided volume was analyzed. The reported accuracy of the system was ±0.01 mL/s of Q_max_ and ±0.17 mL of Vvoided. The graphic representation of the flow and voided fluid volume is shown in Figure 5.

## 5. Discussion

The present manuscript describes a novel, portable, low-cost, and weight-transducer-based home-based UF. A numerical novel algorithm helps to filter the dynamic effect due to urine gravity acceleration during the fall into the vessel placed on the load cell (gravity effect), simplifying the home measurement procedure by removing the funnel. The funnel absence and the algorithm development are the main differences compared to the office-based setting and has helped the development of a comfortable and user-friendly tool. High recorded data accuracy and repeatability have been demonstrated using calibrated water flow. Although a real-world setting validation is required for clinical application, the preliminary data are encouraging.

In addition to the developing interest in telemedicine and telehealth, this approach based on domiciliary tests and an online platform can revolutionize the urologic clinic landscape by eliminating the time wasted waiting in an office setting. Telemedicine has evolved into a wide range of forms and uses electronic devices to improve healthcare delivery in several contexts [13]. This new technology has the potential to improve patient care while reducing health care costs and acquires more importance considering the social distancing that is becoming the “new normal” due to the COVID-19 pandemic. Our approach could be considered a cost-effective and resource-efficient strategy that protects both patients and healthcare providers while ensuring the care of vulnerable patients.

Furthermore, the management of men with LUTS due to BPO is driven by symptom status, risk of disease progression, and the presence of BPH-related complications, such as recurrent urinary retention, urinary tract infection hydronephrosis, and decline in renal function. EAU guidelines suggest a risk-adapted, individualized approach. In men with mild symptoms (IPSS < 8), no complications, and low risk of disease progression, a conservative, watchful waiting approach is recommended [14]. In patients with moderate/severe symptoms and no complications, medical therapy is indicated [1,15,16,17,18]. Even if is beyond the scope of this article to describe the various treatment options [19,20], it is evident that constant and close objective monitoring of LUTS symptoms and therapeutic follow-up is highly needed [21,22,23,24,25]. Our home assessment device surely permits us to reach these goals better than the office-based one by offering the potential to provide cost-effective, high-quality, and constantly up-to-date data allowing for clinical decisions.

Although currently, office UF plays a key role in the evaluation of voiding disorders, a single UF examination is not considered satisfactory to provide an accurate picture of a patient’s BPO because many patients are not able to relax and because circadian rhythms influence UF parameters. Moreover, significant differences in Qmax and Vvoid were seen according to time of day as reported by Porru et al., who found significant circadian differences in multiple measurements of Q_max_ [26]. For this reason, therapeutic decisions made on a single-flow examination can be questionable [27]. Although UF is considered a noninvasive test, it is influenced by the environment and patient’s emotional distress. In this scenario, home-based UF may accomplish these limits with repetitive and comfortable measurements.

This study shows some limitations. Although the correlation between urine flow rate and predicted flow rate in a laboratory setting was satisfactory with a reported accuracy of ±0.01 mL/s and ±0.17 mL of Q_max_ and V_voided_, respectively, the need to test the device on a patient cohort is required. Moreover, the absence of real-world setting validation is certainly needed to determine whether there is a pattern of discordance between the office-based and home-acquired UF. Certainly, further studies are required, and this aspect will be assessed in further papers. However, we strongly believe that the described device with its patented technology and laboratory-setting validation is worthy of interest for a big audience. Furthermore, a disadvantage of home-based UF is the impossibility to assess PVR, which is part of the guideline recommendation. A possible solution could be to perform a first clinic visit to determine PVR evaluation. Further studies are required to confirm quantitative data and to validate this home-based UF over the office-based one.

## 6. Conclusions

Within the exponential growth of telemedicine, this novel and compact home-UF demonstrated, in a laboratory setting, its reliability and promises together with an upcoming real-world validation to be a useful and invaluable tool in routine urological practice, allowing for repeated daily measurements in a comfortable home environment. Through a dedicated online platform, the physician will be able to monitor the health status of the users and customize remotely and in real-time therapies.

## Figures and Tables

**Table 1:** Cost analysis: (a) Consumables; (b) Component costs.

**(a) Consumables.**	
**Consumables**	**Cost (EUR)**
ADC HX711 load Cell	6.99
Node MCU ESP8266 ESP-12F	11.99
waterproof button switch (2 mm round)	0.82
Oled Display I2C IIC0.96 inich	6.99
DC Jack	0.72
CEM 1201	0.69
weipu sp 12 series	15.43
Pedrini Jug 1lt	10.3
IP66 waterproof box	14.29
**(b) Component costs**	
	**Cost (EUR)**
Software	20 *
Server	12 *
Hardware	68.22

* These costs can be further amortized as the number of produced devices increases.

**Table 2:** Repeatability Test.

	Q_max_ (mL/s)	T_total_ (s)	Q_ave_ (mL/s)	V_voided_ (mL)
Test 1	4.35	53.7	3.57	191.9
Test 2	4.36	53.7	3.57	192.0
Test 3	4.37	53.7	3.57	192.1
Test 4	4.34	53.7	3.56	191.6
Test 5	4.35	53.7	3.57	191.9
Test 6	4.35	53.7	3.57	191.9
Test 7	4.35	53.7	3.57	191.9
Test 8	4.35	53.7	3.57	191.9
Test 9	4.35	53.7	3.57	191.9
Test 10	4.36	53.7	3.57	192.0
Test 11	4.37	53.7	3.57	192.1
Test 12	4.34	53.7	3.56	191.6
Test 13	4.37	53.7	3.57	192.1
Test 14	4.34	53.7	3.56	191.6
Test 15	4.35	53.7	3.57	191.9
Test 16	4.35	53.7	3.57	191.9
Test 17	4.35	53.7	3.57	191.9
Test 18	4.35	53.7	3.57	191.9
Test 19	4.37	53.7	3.57	192.1
Test 20	4.34	53.7	3.56	191.6
Test 21	4.35	53.7	3.57	191.9
Test 22	4.36	53.7	3.57	192.0
Test 23	4.35	53.7	3.57	191.9
Test 24	4.37	53.7	3.57	192.1
Test 25	4.34	53.7	3.56	191.6
Test 26	4.35	53.7	3.57	191.9
Test 27	4.36	53.7	3.57	192.0
Test 28	4.37	53.7	3.57	192.1
Test 29	4.34	53.7	3.56	191.6
Test 30	4.37	53.7	3.57	192.1
Mean	4.35	53.70	3.57	191.89
SD	0.010	0.000	0.004	0.171

Maximum flow rate (Q_max_); time to void in seconds (T_total_); average flow rate (Q_ave_); voided volume (V_voided_); standard deviation (SD).

## Data Availability

Not applicable.

## Supplementary Materials

The following supporting information can be downloaded at: https://www.mdpi.com/article/10.3390/ijerph20043287/s1, Table S1. Gravity term error estimation. Error (in percentage) due to the gravity term is calculated from Equation (3) for different values of volume (2, 10, 100, and 300 mL), flow (1, 5, 10, 15 mL/sec) and fall height (20 (A), 40 (B), and 60 (C) cm, which means an average height of 140, 170, and 200 cm, respectively).
