# Supplementary material for: A Novel Low-Cost Uroflowmetry for Patient Telemonitoring
Source: Int J Environ Res Public Health. 2023 Feb 13;20(4):3287. doi: 10.3390/ijerph20043287 (PMC9960409; doi:10.3390/ijerph20043287)
Supplement: Supplementary file 1 [file ijerph-20-03287-s001.zip › ijerph-2048153-supplementary.pdf]

Table S1. Gravity term error estimation. Error (in percentage) due to the gravity term is calculated from Equation (3) for different values of volume (2, 10, 100, and 300 ml), flow (1, 5, 10, 15 ml/s) and fall height [20 (A), 40 (B), and 60 (C) cm. Which means an average height of 140, 170, and 200 cm, respectively).

A				B				C			
h= 20 cm				h= 40 cm				h= 60 cm			
Measured volume V_m (ml)	Measured Flow rate (ml/s)	Effective volume V (ml)	Difference V_m-V (in %)	Measured volume V_m (ml)	Measured Flow rate (ml/s)	Effective volume V (ml)	Difference V_m-V (in %)	Measured volume V_m (ml)	Measured Flow rate (ml/s)	Effective volume V (ml)	Difference V_m-V (in %)
2	1	1.96	2.0	2	1	1.92	4.1	2	1	1.88	6.1
10	1	9.96	0.4	10	1	9.92	0.8	10	1	9.88	1.2
100	1	99.96	0.0	100	1	99.92	0.1	100	1	99.88	0.1
300	1	299.96	0.0	300	1	299.92	0.0	300	1	299.88	0.0
2	5	1.80	10.2	2	5	1.59	20.4	2	5	1.39	30.6
10	5	9.80	2.0	10	5	9.59	4.1	10	5	9.39	6.1
100	5	99.80	0.2	100	5	99.59	0.4	100	5	99.39	0.6
300	5	299.80	0.1	300	5	299.59	0.1	300	5	299.39	0.2
2	10	1.59	20.4	2	10	1.18	40.8	2	10	0.78	61.2
10	10	9.59	4.1	10	10	9.18	8.2	10	10	8.78	12.2
100	10	99.59	0.4	100	10	99.18	0.8	100	10	98.78	1.2
300	10	299.59	0.1	300	10	299.18	0.3	300	10	298.78	0.4
2	15	1.39	30.6	2	15	0.78	61.2	2	15	0.16	91.8
10	15	9.39	6.1	10	15	8.78	12.2	10	15	8.16	18.4
100	15	99.39	0.6	100	15	98.78	1.2	100	15	98.16	1.8
300	15	299.39	0.2	300	15	298.78	0.4	300	15	298.16	0.6
